# The accuracy of geostatistics for regional geomagnetic modeling in an archipelago setting

**DOI:** 10.1038/s41598-022-10362-1

**Published:** 2022-05-06

**Authors:** Muhamad Syirojudin, Eko Haryono, Suaidi Ahadi

**Affiliations:** 1 Doctoral Program in Geography, Faculty of Geography, Gadjah Mada University, Yogyakarta, Indonesia; 2 Department of Environmental Geography, Gadjah Mada University, Yogyakarta, Indonesia; 3 Meteorological, Climatological, and Geophysical Agency, Jakarta, 10720 Indonesia

**Keywords:** Geomagnetism, Tectonics

## Abstract

Indonesia as an archipelago country relies on a limited number and clustered distributed repeat station networks. This paper explores the use of geostatistical modeling to overcome this data limitation. The model data set consisted of repeat station data from 1985 to 2015 epoch. The geostatistical methods utilized included ordinary kriging (OK), collocated cokriging (CC), and kriging with external drift (KED). The model generated using these geostatistical methods was then compared to spherical cap harmonic analyses (SCHA) and polynomial models. The geostatistical model was shown to perform better, with greater accuracy in declination, inclination, and total intensity, as indicated by the root mean square error (RMSE). We have demonstrated that the geostatistical method is a promising approach in the modeling of regional geomagnetic field, especially in areas with limited and clustered distributed data.

## Introduction

The geomagnetic field is dynamic as a result of fluid and material movement in the earth's core, mantle, and crust^1,2^. Therefore, periodic observation and modeling are needed to produce up-to-date geomagnetic field maps. An isogonic chart of declination components was made for the first time by Halley in 1701^3^, and Gauss initiated the global geomagnetic field model in 1832 based on spherical harmonic analysis^4^. Modern global geomagnetic field mapping began in 1957 at the International Union of Geodesy and Geophysics (IUGG) meeting in Toronto. The conference marked the birth of the World Magnetic Survey^5^. In 1960, a mathematical model based on spherical harmonic analysis was recommended. By international agreement, the International Geomagnetic Reference Field (IGRF) model^6,7^ of the geomagnetic field and its secular variations^8^ was thus established.

The IGRF model is updated every five years with oversight from the International Association of Geomagnetism and Aeronomy (IAGA). Improvements to the IGRF model’s accuracy have been achieved by incorporating satellite data and adding the truncation order of the spherical harmonic analysis equation. However, there are still limitations involving accuracy and spatial resolution^9^. These limitations are caused by the truncation order of the spherical harmonic analysis equation^7^, asymmetric distribution of repeat stations and geomagnetic observatories^10^, and the inability to model lithospheric geomagnetic anomaly due to its long spectrum^11^. Comparing IGRF and regional geomagnetic data in China shows that the difference in total intensity can reach 143 nT^12^. For research and exploration, geomagnetic data require an accuracy of 0.1° in the declination component and 50 nT in the total intensity^13^. Furthermore, the spherical harmonic analysis equation cannot be used for a geomagnetic field in the form of an orthogonal area^14^. This has led to the introduction of new regional geomagnetic field models in the hope of producing a more accurate model.

**Figure 1:**
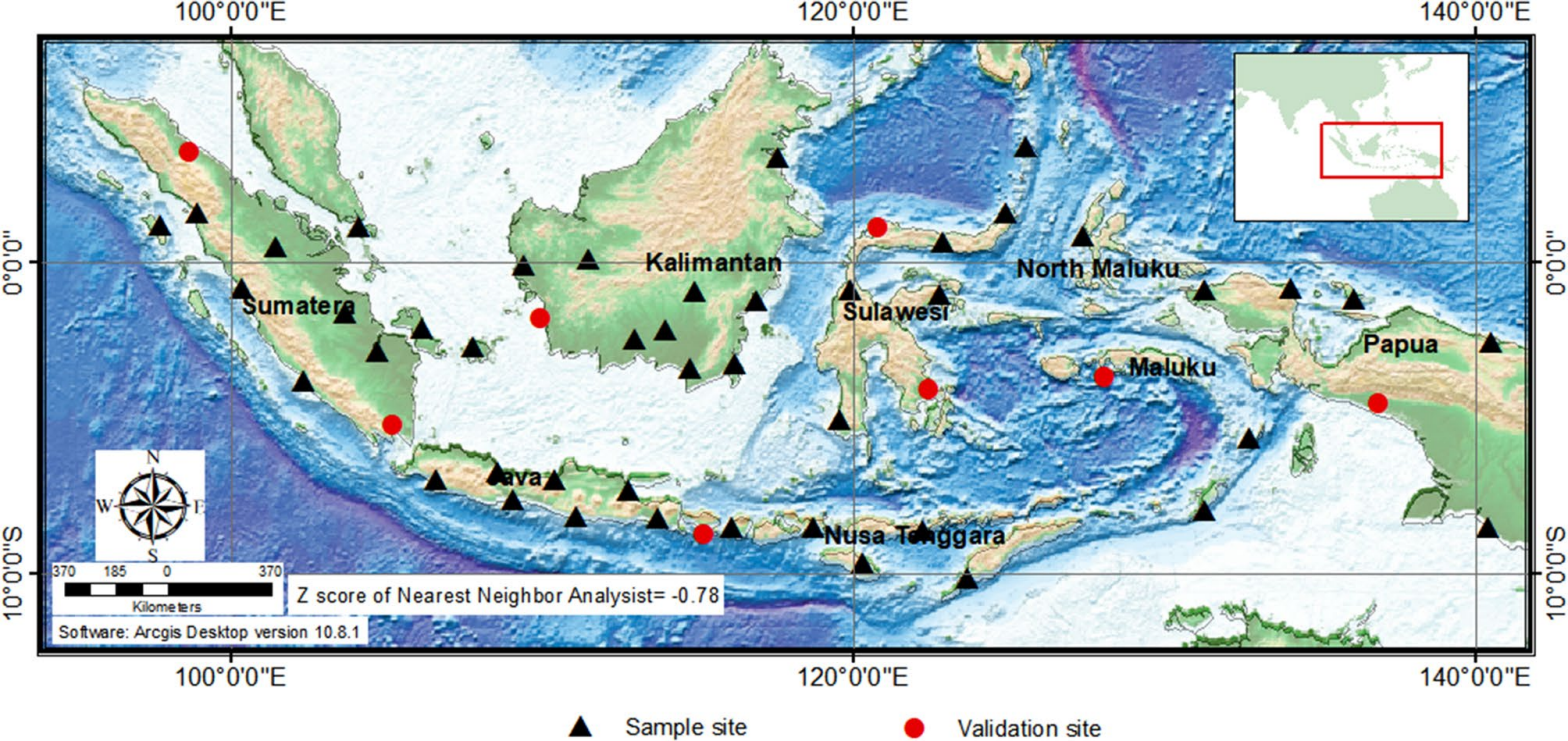
Distribution of the 45 sample locations and the 8 locations designated for data validation, Epoch 2010. The Z score is the nearest-neighbor analysis value; a negative score indicates cluster distribution. The base map is from DEMNAS (the national digital elevation model) publish by the National Mapping Agency of Indonesia (https://tanahair.indonesia.go.id/demnas/#/). The national bathymetry is generated from the inversion of gravity anomaly data incorporated with additional single and multibeam sounding by the National Mapping Agency of Indonesia, National Geophysical Data Center, British Oceanographic Data Centre, Agency for the Assessment and Application of Technology Indonesia, Indonesian Institute of Sciences, and Research and Development Center for Marine Geology of Indonesia.

Based on IAGA Resolution No. 23 (1963), “Repeat station measurements during the International Years of the Quiet Sun (IQSY) in support of the World Magnetic Survey (WMS),” the Badan Meteorologi, Klimatologi dan Geofisika (BMKG), or the Meteorological, Climatological, and Geophysical Agency of Indonesia have conducted measurements in repeat stations to cover the region of Indonesia starting in 1985. These measurements were routinely carried out every five years. The locations of the Indonesian repeat stations comply with some of the criteria set forth by the IAGA^15^. The cluster distribution of the repeat stations of the Indonesian archipelago is characterized by nearest-neighbor analysis with a negative value (Z score = −0.78). Repeat station distribution is difficult to make uniform^16^. The repeat station locations cannot be placed on the water, as those sites cannot be accessed. No locations were found that met the standards for repeat stations^17^. This situation problematizes the regional geomagnetic field modeling framework in Indonesia (Fig. 1).

The methods generally used in regional geomagnetic field modeling include polynomial^18,19^, spline, Taylor expansion^20,21^, spherical cap harmonic analysis (SCHA)^22^, revised spherical cap harmonic analysis^23^, and revised spherical cap harmonic analysis for the Earth’s surface^24^. These methods typically require dense data for modeling. The boundary effects of the existing methods are also affected by the data configuration. Numerical instability due to wide data gaps resulting from sparse data patterns needs to be balanced with statistical or physical regulation to increase model reliability^25^.

We propose the geostatistical method as an alternative for regional geomagnetic field modeling using limited and clustered data patterns. A geostatistical method predicts the value of data property which is not covered by the sample data distribution or a sparse data pattern^26^. Originally developed to predict the distribution percentages of ores for mining^27^, geostatistical methods also very accurately model natural resources^28^ such as underground rivers^29^ and reservoirs of oil and gas^30,31^.

Geostatistical methods are classified into two types of algorithms: traditional and geostatistical^32^. The weighted values in the traditional algorithm are based on the geometrical distance of the surrounding estimator data. This includes inverse distance weighting (IDW), nearest neighbor or polygon, and triangulation methods. Geostatistical algorithms, on the other hand, use the structure or statistics of distances from the surrounding estimator data to weigh the data property. The geostatistical kriging algorithm evaluates the distance and direction of the array of sample data points to reflect spatial correlations. Kriging can produce good estimates for anisotropic data and clustered distributions^33^. There are many kinds of kriging algorithms: e.g., simple kriging, ordinary kriging (OK)^34,35^, universal kriging, kriging with external drift (KED)^36^, and collocated cokriging (CC)^37^.

**Figure 2:**
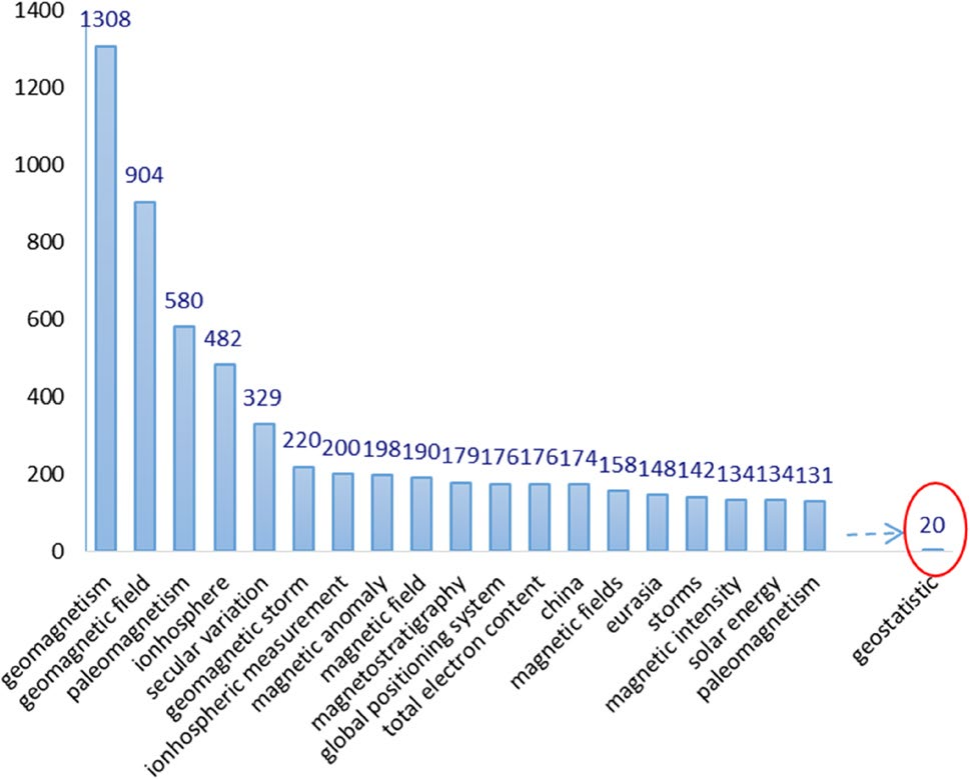
The occurrences of words in the titles, keywords, and abstracts of the 3,524 Scopus-indexed papers using the keywords “geomagnetic” and “regional.” The keyword “geostatistical” has 20 occurrences. However, out of those 20, only 3 papers discuss geomagnetic regional modeling.

However, geostatistical methods are rarely used for regional geomagnetic field modeling. A search on Scopus for indexed papers using the keywords "geomagnetic” and “regional" resulted in only 3 of 3524 papers referencing geomagnetic regional modeling (Fig. 2). No paper discusses the geostatistical method for geomagnetic regional modeling with special emphasis on clustered distributed data. Accordingly, the purpose of this paper is to explore the accuracy of the geostatistical method in regional geomagnetic field modeling and mapping.

## Results

**Figure 3:**
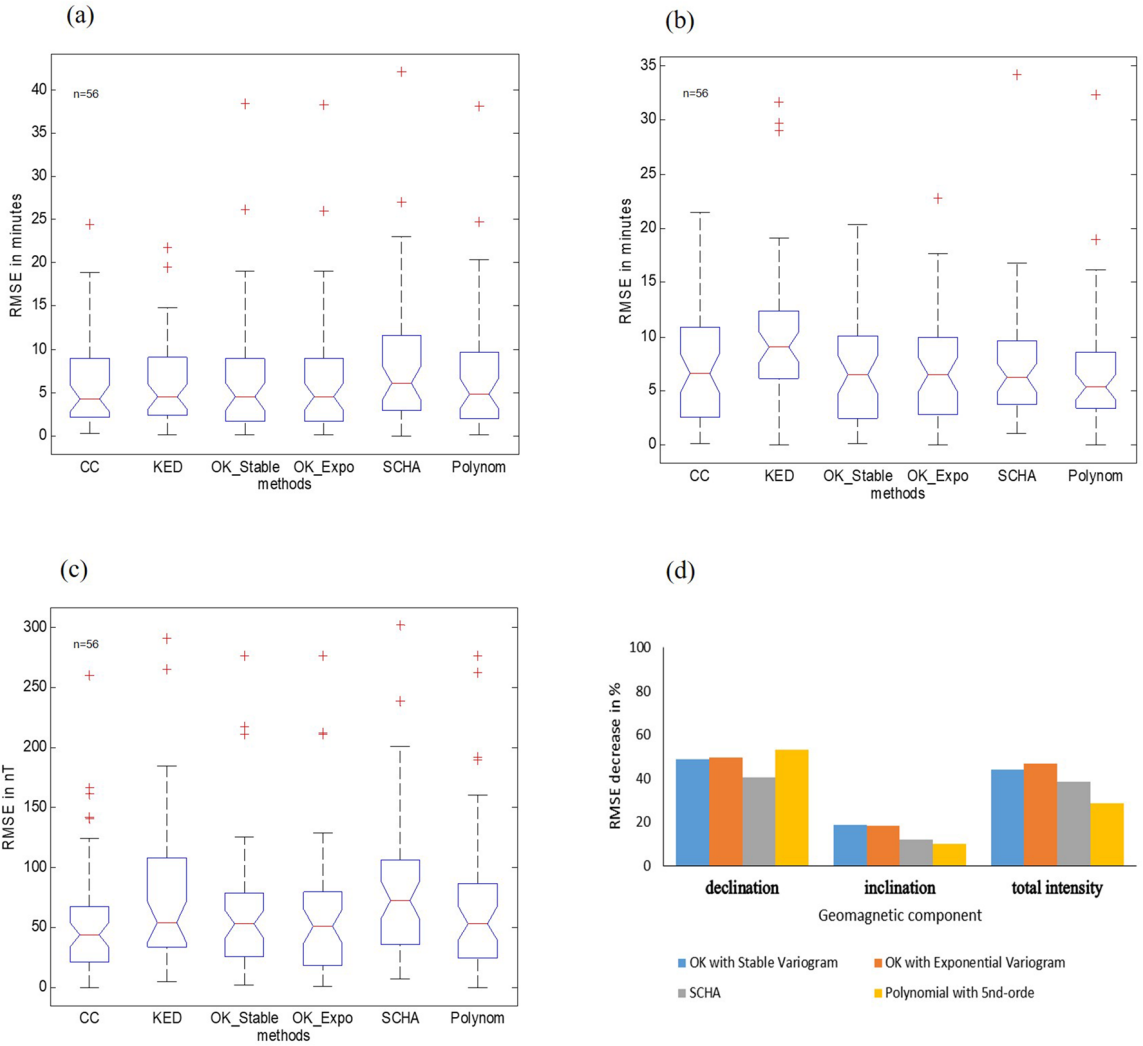
Boxplot of root mean square error (RMSE) (*n* = 56 data) between data estimation and validation from the CC, KED, OK methods with stable variogram, OK with exponential variogram, SCHA, and polynomial methods data for epoch 1985– 2015 for each geomagnetic component. (**a**) Declination Component (minutes); (**b**) Inclination Component (minutes); (**c**) Total Intensity (nT); (**d**) RMSE decreased below normalized value due to the addition of 28% more sample data.

The geostatistical method generally yields a smaller root mean square error (RMSE) than do other existing methods for regional geomagnetic modeling in the Indonesian archipelago. The smallest RMSE for the declination component (7.03 minutes) is yielded by the collocated cokriging (CC) method and an identical range of 2.048.97 minutes using both KED and OK (Fig. 3a). The KED method yields a slightly higher RMSE of 7.19 minutes. The OK method, using stable and exponential variograms, yields RMSE values of 7.67 and 7.68 minutes, respectively. On the other hand, the polynomial and SCHA methods show an RMSE of 7.96, and 9.26 minutes, and have widerange values of 1.93–9.67, and 2.9311.97 minutes, respectively.

However, for the inclination component, not all geostatistical methods yield small RMSE values. As shown in Fig. 3b, the smallest RMSE is returned using OK, with a stable variogram (6.77 minutes). This is followed by OK with an exponential variogram (6.90 minutes) and has range values of 2.6210.83 minutes. CC has a higher RMSE than the polynomial method, and KED has a higher RMSE than both the polynomial and SCHA methods. The polynomial method has an RMSE of 6.91 minutes with a range of 3.448.55 minutes, the CC has an RMSE of 7.19 minutes and has an identical range with OK, the SCHA has an RMSE of 8.15 minutes with a range of 3.789.63 minutes, and the KED has an RMSE of 10.28 minutes with a range of 6.1112.36 minutes.

As expected, for total intensity the geostatistical method yields smaller RMSE values as shown in Fig. 3c. The smallest RMSE is obtained by the CC method (57.07 nT) with the shortest range of 21.6567.75 nT, followed by OK (63.58 nT) with a range of 25.6078.20 nT. On the other hand, KED (74.54 nT) has a higher RMSE than the polynomial method (68.97 nT), but smaller than SCHA (82.24 nT).

## Discussion

The results show that the geostatistical method is a good model of the regional geomagnetic field based on data from typical clustered distribution patterns, as exemplified in Indonesia. The geostatistical model using CC was 2.23 minutes more accurate than the SCHA and polynomial models of the declination component. The CC model also was 25.17 nT more accurate than the SCHA and the polynomial models of the total intensity component. However, for the inclination component, the OK model was more accurate (0.43 minutes smaller) than the polynomial and SCHA models. The dataset in Indonesia consists of the archipelago’s clustered data with average distances among the repeat stations of 250 km (Fig. 1). This distance and distribution are not spherical caps enough. The distance among the repeat station points is quite large, as the sea forms a gap among the repeat stations. The large distances between the sample data causes the accuracy of the SCHA to be lower^38,39^ because additional statistical or physical regulation are required to compensate for the numerical instability^25^. The same problem occurs with the polynomial method, though typically this method will produce good accuracy if the data distribution is uniform^16^.

The RMSE values of the geomagnetic regional modeling parameters are affected by the spatial distribution of the repeat stations. The same phenomenon was also reported in China^40^, where a randomly distributed pattern produced a noticeable effect. Importantly, the SCHA and polynomial methods do not produce effective models based on clustered distributed patterns. Both these methods require more regularly and densely distributed data^25,41,42^. That the CC and OK methods might better fit a wider range of data distributions was also suggested by Webster and Oliver^26^ and Zhao et al.^43^. The CC and OK methods are suitable for random, clustered, and anisotropic distributions^26,43^. They are also able to deal with different weights between clusters using varied numbers of data sets, which can reduce bias in the estimations^32^. Meanwhile, the SCHA and polynomial methods cannot accept a different number of data sets between clusters.

**Figure 4:**
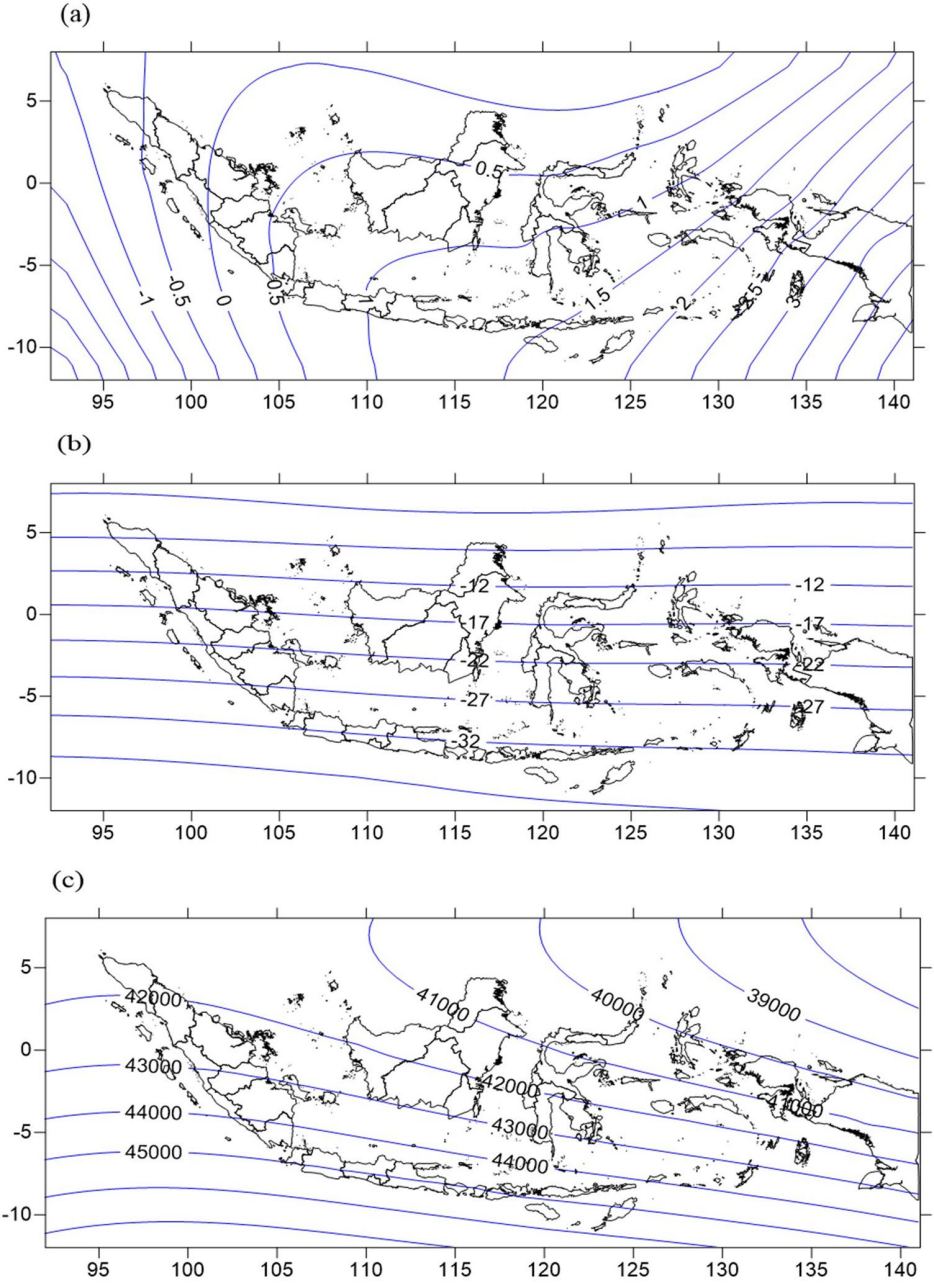
Geomagnetic chart for epoch 2015.0 using the collocated cokriging method in Indonesia; (**a**) Declination Comp. with Δ = 0.5°, (**b**) Inclination Comp. with Δ = 5°, and (**c**) Total Intensity Comp. with Δ = 1000 nT.

The average RMSE of the inclination component from OK is slightly lower than with the polynomial method (0.13 minutes). For the declination and total intensity components whose contours are a curving isoline, the CC method produces a smaller average RMSE than the OK, SCHA, and polynomial methods. This is ideal for the curving isoline of geomagnetic components that results from additional secondary data (IGRF-13). This result was also in agreement with the work of Rivoirard^37^ and Han et al.^44^ that showed CC could result in a lower RMSE for complex data property. The larger RMSE of the SCHA and polynomial methods probably results from the curving isoline of the geomagnetic declination. Indonesia, which lays in the equator, always has a banding isoline; the declination’s contours in Indonesia deviate along the longitude (Fig. 4). That the SCHA and polynomial methods have a larger RMSE with a curving isoline was also found by Gu et al.^45^ in China and in Europe by Korte et al.^11^. The polynomial method is more accurate for a straight geomagnetic isoline^46^.

Our results also reveal that when adding more repeat station data sets, the geostatistical method results in better increment accuracy than the SCHA and polynomial methods. The research used the 2015 data set (with 28% additional sample data), compared to the data set from 1990 to 2010 (Fig. 3d). The accuracy of the OK method increased by 47% for the total intensity component. This increment of OK accuracy is 18% higher than that of the SCHA and polynomial methods. The accuracy of OK also increases by 19% for the inclination component, which is 9% higher than SCHA and polynomial values. For the declination component, the accuracy of OK also increases by 50%, or 9% higher than SCHA, but a bit lower (3%) than polynomial. However, the accuracy of the CC and KED methods did not increase as significantly as the OK method. The use of a secondary variable^36,37,47,48^ in the CC and KED methods may account for the smaller increase. This result also showed that with additional data sets, the SCHA and polynomial methods do not result in an accuracy increase as significant as those seen in previous research from Europe^49,50^, where the data set was regularly distributed.

## Methods

We used repeat station data from 1985 to 2015 published by BMKG, and the Indonesian authority granted permission to conduct geomagnetic measurements under the IAGA resolution. Most of the repeat stations are located at the airports of the Indonesian islands. This has the advantage that, in addition to fulfilling the special requirements for the location of the repeat stations, the stations can also support the calibration of runway azimuth, which is important for aircraft navigation^51,52^. These measurements were then routinely carried out every five years. The data has been reduced by diurnal and secular variation to get the same time for every period.

To validate the regional geomagnetic model produced by the geostatistical methods (OK, CC, and KED) and the geomagnetic methods (SCHA and polynomial), we used 15% of the total existing repeat station data gathered from 1985 to 2015. The data used for model validation purposes was excluded from the modeling process. The composition and distribution of the validation data were selected randomly (Fig. 1). For the main geomagnetic field, we used the Definitive Geomagnetic Reference Field (DGRF) data generated from IGRF-13, since the global model is more accurate than the regional one^53^.

This research tested the accuracy of the geostatistical method and the existing methods to model the regional geomagnetic field within the Indonesian territory. From the geostatistical method group, we used the OK, CC, and KED methods. The OK equation is as follows^32^:

1 $$ Z_{{{\text{OK}}}} (x_{0} ) = \sum\limits_{i = 1}^{n} {\lambda_{i} } Z(x_{i} ). $$

where *Z*_*OK*_ is the data estimation result, λ is the weight of each primary data value, and Z(xi) is the data value in the ith location. This method is easily applied and considered the best linear unbiased estimator (BLUE). This method has been used in many fields of earth science^54–57^ and produces reasonable estimates.

The CC equation is as follows^37^:

2 $$ Z_{CC} (x_{0} ) = \sum\limits_{i = 1}^{n} {\lambda_{i} } Z_{1} (x_{i} ) + \mu Z_{2} (x_{0} ). $$

where *Z*_*CC*_ is the data estimation result, λ is the weight of the measurement datum in the ith location, Z_1_ is the measurement datum in the ith location, μ is the weight of the available complementary datum from IGRF-13, and Z_2_ is the available complementary datum from IGRF-13.

The KED equation is as follows^36^:

3 $$ Z_{KED} (x_{0} ) = \sum\limits_{i = 1}^{n} {\lambda_{i} } Z_{1} (x_{i} ) + a\sum\limits_{i = 1}^{n} {\lambda_{i} } Z_{2} (x_{i} ) + aZ_{2} (x_{0} ). $$

where Z_*KED*_ (*x*_*0*_) is the estimation result in location *x*_*0*_, *Z*_*1*_ (*x*_*i*_) is the measurement datum from the BMKG in the ith repeat station, *λ*_*i*_ is the weight of *Z*_*1*_ (*x*_*i*_) and *Z*_*2*_ (*x*_*i*_), and *a* is the slope of the available complementary data from IGRF-13 in location *x*_*0*_.

The geostatistical method was then compared with the SCHA and polynomial methods. The SCHA method was chosen because it has been used frequently by researchers in recent decades^25^. The potential field equation V in the SCHA method is the following:

4 $$ \begin{aligned} V(r,\theta ,\lambda ) = & a\sum\limits_{k \ge 0}^{{k_{i} }} {\sum\limits_{m \ge 0}^{k} {\left( \frac{a}{r} \right)^{{n_{k} (m) + 1}} \left( {g_{{n_{k} }}^{i,m} \cos (m\lambda ) + h_{{n_{k} }}^{i,m} \sin (m\lambda )} \right)P_{{n_{k} }}^{m} \theta } } \\ & \;\; + a\sum\limits_{k \ge 0}^{{k_{e} }} {\sum\limits_{m \ge 0}^{k} {\left( \frac{r}{a} \right)^{{n_{k} (m)}} \left( {g_{{n_{k} }}^{e,m} \cos (m\lambda ) + h_{{n_{k} }}^{e,m} \sin (m\lambda )} \right)P_{{n_{k} }}^{m} \theta } } . \\ \end{aligned} $$

where V = geomagnetic potency, a = earth’s radius (6,371.2 km), r = radial distance of the set location from the Earth’s core, *θ* = colatitude (90°–latitude), λ = longitude, $P_{{n_{k} }}^{m} \theta$ = Legendre function of the associated Schmidt non-integer *n*_k_(*m*) and *m*, $g_{{n_{k} }}^{i,m}$ and $h_{{n_{k} }}^{i,m}$ are the Gaussian Earth’s core magnetic field coefficients, $g_{{n_{k} }}^{e,m}$ and $h_{{n_{k} }}^{e,m}$ are the Gaussian electric current coefficients.

The polynomial method was also selected for comparison because it is usually more accurate for regional geomagnetic modeling^45,58^. The Taylor polynomial normal field model equation is given below^14,45,58^:

5 $$ C(\theta ,\varphi ) = \sum\limits_{n = 0}^{N} {\sum\limits_{m = 0}^{n} {A_{nm} } } (\theta - \theta_{0} )^{n - m} (\varphi - \varphi_{0} )^{m} . $$

where *C* is the geomagnetic field component, A_*nm*_ is the Taylor polynomial coefficient, *θ* is the latitude, *ϕ* is the longitude, and *θ*_*0*_ and *ϕ*_*0*_ are the latitude and longitude from the center of the region.

The data from the repeat stations was cut by the diurnal variation from BMKG observatories. The data was then checked and refined by applying a secular variation. After the data has the same time epoch (e.g., epoch 2015.0), geomagnetic data (excluding the 8 validation locations) were used to estimate the geomagnetic values of the validation locations for the CC and KED methods.

For the OK, SCHA, and polynomial methods, the geomagnetic data for each component X, Y, and Z were decremented with the DGRF data to calculate the geomagnetic field anomaly. The short spectrum of the SCHA method was not suitable to model the main geomagnetic field^38^. Mathematically, the procedure was formulated in Eqs. (6), (7), and (8):

6 $$ \Delta X = X - {\text{DGRF\_X}} $$

7 $$ \Delta Y = Y - {\text{DGRF\_Y}} $$

8 $$ \Delta Z = Z - {\text{DGRF\_Z}} $$

where ΔX, ΔY, and ΔZ are the geomagnetic field anomaly of each component and DGRF_X, DGRF_Y, and DGRF_Z are the main geomagnetic definitive data obtained from the IGRF-13 model for each component.

**Figure 5:**
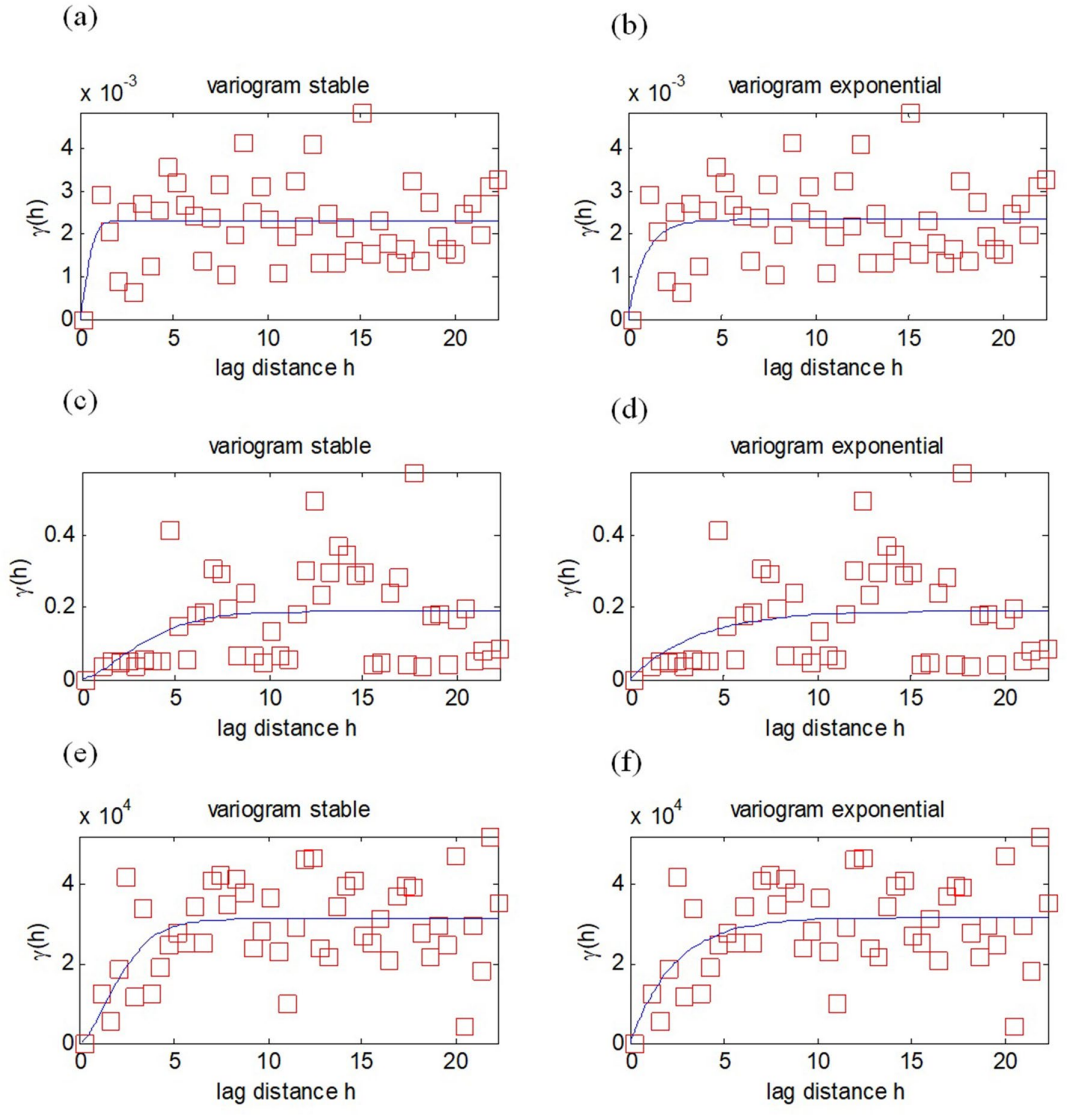
Semivariogram of geomagnetic data from epoch 2010 for the OK method. Clustered overlapping experimental variograms as characteristic of the clustered distributed data pattern, resulting in identical theoretical variogram parameters between stable and exponential. (**a**) stable variogram of declination comp., (**b**) exponential variogram of declination comp., (**c**) stable variogram of inclination comp., (**d**) exponential variogram of inclination comp., (**e**) stable variogram of total intensity comp., and (**f**) exponential variogram of total intensity comp.

The number of sample data sets and locations used for each epoch’s calculation is shown in Table 1. Equation (1) was used for the OK method, Eq. (2) was used for the CC method, Eq. (3) was used for the KED method, Eq. (4) was used for the SCHA method, and Eq. (5) was used for the polynomial method with *θ*_*0*_ = 2° and *ϕ*_*0*_ = 117.5°. The OK method used stable and exponential variograms. The variogram parameters used in the OK model are range 3.41, sill 0.0023, and nugget 0.0001 for the declination component; range 10.2, sill 0.19, and nugget 0.01 for the inclination component; and range 5.95, sill 31,216, and nugget 1 for total intensity (Fig. 5). The selection of the variogram affects the estimation results of the kriging method^26^. The CC and KED used a spherical variogram. For the SCHA method, an 8th-order truncation was used for the geomagnetic anomaly, because the RMSE of the ΔX, ΔY, and ΔZ components for the 8th order was smaller and stable. For the polynomial method, the 5th order was used because it produced a small RMSE value. We then analyzed the estimation data to determine the root mean square error (RMSE). We then used RMSE to compare the accuracy of the geostatistical method in modeling the regional geomagnetic field with the SCHA and polynomial methods.

**Table 1:** The number of sample and validation data sets used in each epoch 1985–2015.

Epoch	Total repeat stations	Number of sample locations (repeat stations)	Number of validation locations
1985	59	50	8
1990	53	45	8
1995	53	45	8
2000	53	45	8
2005	54	46	8
2010	53	45	8
2015	68	58	8

## Data Availability

The geomagnetic data for 1985–2015 used in this study was extracted from BMKG**.** The Definitive Geomagnetic Reference Field (DGRF) data was generated from IGRF-13 (https://www.ngdc.noaa.gov/IAGA/vmod/igrf.html). The analysis software tool used was Matlab (License ID: 1,106,171). Nearest-neighbor analysis to determine the type of data sample distribution was calculated using the Z score (http://ceadserv1.nku.edu/longa//geomed/ppa/doc/NNA/NNA.htm). The geomagnetic regional model epoch 2015 is available at http://doi.org/10.5281/zenodo.5094914.

## Abbreviations

BMKG: Badan Meteorologi, Klimatologi dan Geofisika, or the Meteorological, Climatological, and Geophysical Agency of Indonesia
CC: Collocated cokriging
DGRF: Definitive geomagnetic reference field
IAGA: International association of geomagnetism and aeronomy
IDW: Inverse distance weighting
IGRF: International geomagnetic reference field
IUGG: International union of geodesy and geophysics
IQSY: International years of the quiet sun
KED: Kriging with external drift
OK: Ordinary kriging
SCHA: Spherical cap harmonic analysis
RMSE: Root means square error
WMS: World magnetic survey

## References

[1] Elsasser WM (1956). Hidromagnetic dynamo theory. Rev. Mod. Phys..

[2] Brandenburg A (2007). Hydromagnetic dynamo theory. Scholarpedia.

[3] Cook A (2001). Edmond halley and the magnetic field of the earth. Notes Rec. R. Soc. Lond..

[4] Garland GD (1979). The contributions of Carl Friedrich Gauss to geomagnetism. Hist. Math..

[5] Heppner JP (1963). The world magnetic survey. Space Sci. Rev..

[6] Zmuda AJ (1969). International geomagnetic reference field 1965.0. J. Geomagn. Geoelectr..

[7] Macmillan S, Finlay C (2011). The international geomagnetic reference field. Geomag. Observ. Models.

[8] Mandea M, Macmillan S (2000). International geomagnetic reference field’the eighth generation. Earth Planets Sp..

[9] Maus S (2005). The 10th generation international geomagnetic reference field. Phys. Earth Planet. Inter..

[10] Golovkov VP, Bondar TN, Burdelnaya IA (2000). Spatial-temporal modeling of the geomagnetic field for 1980–2000 period and a candidate IGRF secular-variation model for 2000–2005. Earth Planets Sp..

[11] Korte M, Korte M, Lesur V (2012). Repeat station data compared to a global geomagnetic field model. Ann. Geophys..

[12] Wang T (2003). The analysis of the IGRF error in the China continent. Chin. J. Geophys..

[13] Macmillan S (2004). Earth’s magnetic field. Geophys. Monogr. Ser..

[14] Mandea M, Purucker M (2005). Observing, modeling, and interpreting magnetic fields of the solid earth. Surv. Geophys..

[15] Newitt, L. R., Barton, C. E. & Bitterly, J. *Guide for magnetic repeat station surveys*. (International Association of Geomagnetism and Aeronomy, 1996).

[16] Barraclough, D. R. & Santis, A. D. Repeat station activities. in *Geomagnetic Observations and Models* (ed. M. Mandea, M. K.) 45–56 (Springer Netherlands, 2011). doi:10.1007/978-90-481-9858-0

[17] Newitt, L. R., Barton, C. E., Bitterly, J. & International Association of Geomagnetism and Aeronomy. Working Group V-8: analysis of the global and regional geomagnetic field and its secular variation. *Guide Magn. Rep. Stat. Surv.* **112** (1996).

[18] Düzgit Z, Baydemir N, Malin SRC (1997). Rectangular polynomial analysis of the regional geomagnetic field. Geophys. J. Int..

[19] Alldredge LR (1981). Rectangular harmonic analysis applied to the geomagnetic field. J. Geophys. Res..

[20] Hall CA, Meyer WW (1976). Optimal error bounds for cubic spline interpolation. J. Approx. Theory.

[21] Wahba, G. *Spline Models for Observational Data*. (Society for industrial and applied mathematics, 1990). doi:10.1137/1.9781611970128

[22] Haines GV (1985). Spherical cap harmonic analysis. J. Geophys. Res..

[23] Thébault E, Schott JJ, Mandea M, Hoffbeck JP (2004). A new proposal for spherical cap harmonic modelling. Geophys. J. Int..

[24] Thébault E (2008). A proposal for regional modelling at the Earth’s surface, R-SCHA2D. Geophys. J. Int..

[25] Torta JM (2020). Modelling by spherical cap harmonic analysis: a literature review. Surv. Geophys..

[26] Webster, R. & Oliver, M. A. *Geostatistics for environmental scientists*. (Wiley, 2008). doi:10.1002/9780470517277

[27] Krige DG (1951). A statistical approach to some basic mine valuation problems on the Witwatersrand. J. Chem. Metall. Min. Soc. S. Afr..

[28] Goovaerts, P. *Geostatistics for natural resources and evaluation*. (Oxford University Press, 1997).

[29] Huysmans M, Dassargues A (2009). Application of multiple-point geostatistics on modelling groundwater flow and transport in a cross-bedded aquifer (Belgium). Hydrogeol. J..

[30] Poon DC, McCormack M, Thimm HF (1993). The application of fractal geostatistics to oil estimates. J. Can. Pet. Technol..

[31] Xi Z, Morgan E (2019). Combining decline-curve analysis and geostatistics to forecast gas production in the Marcellus shale. SPE Reserv. Eval. Eng..

[32] Isaaks, E. H. & Srivastava, R. M. *Applied geostatistics*. (Oxford University Press, 1989).

[33] Goovaerts, P. Geostatistics for natural resource evaluation. in *Technometrics* **42**, (1997).

[34] Wackernagel, H. Ordinary Kriging. in *Multivariate geostatistic* 74–81 (Springer, 1995). doi:10.1007/978-3-662-03098-1_11

[35] Montero, J.-M., Gema, F.-A. & Mateu, J. *Spatial and Spatio-Temporal Geostatistical Modeling and Kriging*. (Wiley, 2015).

[36] Hengl T, Heuvelink G, Stein A (2003). Comparison of kriging with external drift and regression-kriging. ITC Techn. Note Enschede Netherlands.

[37] Rivoirard J (2001). Which models for collocated cokriging?. Math. Geol..

[38] Thébault E, Gaya-Piqué L (2008). Applied comparisons between SCHA and R-SCHA regional modeling techniques. Geochem. Geophys. Geosyst..

[39] Schott, J.-J. & Thébault, E. Modelling the earth’s magnetic field from global to regional scales. in *Geomagnetic Observations and Models* 229–264 (Springer Netherlands, 2011). doi:10.1007/978-90-481-9858-0_9

[40] Chen D-X, Liu D-Z, Zeng X-N, Meng L, Yang X-J (2016). Application and improvement of spatial temporal Kriging in geomagnetic field interpolation. Acta Geophys. Sin..

[41] Bonito A, DeVore R, Guignard D, Jantsch P, Petrova G (2021). Polynomial approximation of anisotropic analytic functions of several variables. Constr. Approx..

[42] Korte M, Thébault E (2007). Geomagnetic repeat station crustal biases and vectorial anomaly maps for Germany. Geophys. J. Int..

[43] Zhao S, Zhou Y, Wang M, Xin X, Chen F (2014). Thickness, porosity, and permeability prediction: comparative studies and application of the geostatistical modeling in an Oil field. Environ. Syst. Res..

[44] Han F, Zhang H, Guo Q, Wei K, Shang Z (2018). An integrated method for seismic velocity modeling based on collocated cokriging. J. Geophys. Eng..

[45] Gu Z (2006). Geomagnetic survey and geomagnetic model research in China. Earth Planets Sp..

[46] Ryan, T. P. *Modern Regression Methods*. (Wiley, 2008).

[47] Abedi M, Asghari O, Norouzi G-H (2015). Collocated cokriging of iron deposit based on a model of magnetic susceptibility: a case study in Morvarid mine, Iran. Arab. J. Geosci..

[48] Madani, N. Multi-collocated cokriging: an application to grade estimation in the mining industry. in *39th International symposium on Application of Computers and Operations Research in the Mineral Industry, APCOM 2019* (eds. C., M. et al.) 158–167 (CRC Press/Balkema, 2019). doi:10.1201/9780429320774-18

[49] Verbanac G (2007). On regional modeling of the main geomagnetic field. Geofizika.

[50] Kotzé PB, Korte M (2016). Morphology of the southern African geomagnetic field derived from observatory and repeat station survey observations: 2005–2014. Earth Planets Sp..

[51] Rasson, J. L. & Delipetrov, T. *Geomagnetics for aeronautical safety: a case study in and around the Balkans*. *NATO Security through Science Series C: Environmental Security* (Springer, 2006).

[52] Loubser, L. & Newitt, L. *Guide for Calibrating a compass swing base*. (IAGA, 2009).

[53] Talarn À, Pavón-Carrasco FJ, Torta JM, Catalán M (2017). Evaluation of using R-SCHA to simultaneously model main field and secular variation multilevel geomagnetic data for the North Atlantic. Phys. Earth Planet. Inter..

[54] Cǎţeanu M, Ciubotaru A (2020). Accuracy of ground surface interpolation from airborne laser scanning (ALS) data in dense forest cover. ISPRS Int. J. Geo-Inform..

[55] Massimi L, Ristorini M, Astolfi ML, Perrino C, Canepari S (2020). High resolution spatial mapping of element concentrations in PM10: A powerful tool for localization of emission sources. Atmos. Res..

[56] Nistor MM (2020). Investigation of groundwater table distribution using borehole piezometer data interpolation: case study of Singapore. Eng. Geol..

[57] Sunkari ED, Abu M, Zango MS, Lomoro Wani AM (2020). Hydrogeochemical characterization and assessment of groundwater quality in the Kwahu-Bombouaka Group of the Voltaian Supergroup. Ghana. J. Afr. Earth Sci..

[58] Geese A, Korte M, Kotze PB, Lesur V (2011). Southern African geomagnetic secular variation from 2005 to 2009. S. Afr. J. Geol..

